# Therapeutics

**Published:** 1878-11

**Authors:** 


### Therapeutics.

Therapeutic Employment of the Salts of Conia. *Journ. de Médecine*, Sept., 1878, page 417.

Hemlock, of which the alkaloid is conine or cicutine, has long been employed in cancer, scrofula, syphilis and pulmonary or laryngeal affections, accompanied by spasmodic phenomena. Up to the present time this alkaloid has been regarded as exceedingly poisonous, and only given in very small doses. M. Tyryakian has just shown, in a thesis which is an extremely complete physiological and clinical study of conine and its salts, that this substance is much less poisonous than supposed, and moreover that it produces all the effects of hemlock. Very numerous experiments on animals, which have made clear its action, have been conducted in the laboratory of M. Vulpian. The clinical researches have been made principally in the service of M. Audhoui, who for a long time has been occupied with the properties of the bromhydrate of conia, the salt which presents the most advantages, and has found that below 10 centigrams the medicine does not act; he has further seen that tolerance of the medicine is produced rapidly, which requires a rapid increase of dose, and that elimination taking place very rapidly by the kidneys, it was necessary to give it in large doses. When a rapid action is required, hypodermic injections are best, and a remarkable thing is that although conine has a local effect which renders its injection very injurious, the bromhydrate, on the contrary, causes no accident at the point of puncture. A solution of 1 gram of crystallized bromhydrate of conine in 19 grams of distilled water is used, each gram containing 5 centigrams of the salt. The initial dose for injections does not differ much from what is necessary by the stomach, so that one may begin without inconvenience by a dose of 15 centigrams at once. But if the patient appear too susceptible, or too weak, but 10 or even 5 centigrams should be used. The injections must be often renewed, and the dose rapidly increased to keep the patient under the influence of the medicine.

By the stomach the increase of dose is very easy, and one may readily give very large doses, 50 or 60 centigrams and even more in three or four doses, always taking care to habituate the system by smaller doses. Limit in the progression of doses cannot be assigned; the condition of the patient, the effect desired, and other considerations of this order can alone guide the physician. In one case the dose was raised to 1 gram in twenty-four hours, and in four doses, without any evil results. Both pills and syrup were used. The following are some of the formulas employed by M. Tyryakian: Bromhydrate of conine, 1 gram; sugar of milk, 1 gram; mucilage, q. s. To be made into twenty or forty pills, each containing 5 or 2½ centigrams. A syrup may be made with 1 gram of the bromhydrate of conine, and 250

grams of simple or aromatic syrup, a teaspoonful containing 2 centigrams of the salt. For infants, only 25 centigrams of the salt should be used, a teaspoonful then containing 5 milligrams.

For external use 2 grams of the salt in 40 grams of cucumber ointment. It may be combined with bromide of potassium as in the following: Bromide of potassium, 10 grams; bromhydrate of conine, 1 gram; distilled water, 400 grams. Each spoonful of 20 grams containing 50 centigrams of bromide of potassium, 5 centigrams of bromhydrate of conine. In children, experimented upon, the dose did not exceed 2 centigrams of bromhydrate of conine.

TAYUYA.—(*Virg. Med. Month., July*, '78.)

This drug, to which attention was drawn in a card published in the May number of this journal by Dr. Hyde, is the root of a cucurbitaceous Brazilian plant, the *dermophylla pendulina*. Its physiological action is said to be an increase of saliva and of gastric juice, improved appetite and digestion and slight laxation, in small doses; in large doses it causes vomiting, colic, purging, sweating and salivation.

It is a proprietary preparation and two tinctures are prepared by the Italian pharmacists,—"*tra madre*" and "*tra deluta.*" The former is used hypodermically in gram doses; and locally diluted with water. The weaker tincture is made with one part of the former to three parts of rectified spirit, and is given internally in doses two to twenty drops two or three times a day.

The value of the remedy in syphilis is still *sub judice.*

OXIDE OF ZINC IN DIARRHŒA.—(*Bul. de Thérap.*)

Bouany claims that every case of chronic diarrhœa treated with zincic oxide and sodic bicarbonate, results favorably. Moreover, while cases treated in the ordinary way are liable to recur, those handled according to this plan never return.

LIME WATER AND MILK COMPATIBLE WITH CALOMEL.—(*Virg. Med. Month.*, July, 1878.)

At a June meeting of the Richmond Acad. of Med., Dr. Joynes called attention to the above subject, and to his experiments as to whether we could employ these two remedies together without the formation of *black wash* in the stomach. Dr. Chapman had long ago stated that the emulsion of lime-water and milk, was not disturbed by citric or hydrochloric acids, nor was a black precipitate thrown down by calomel. Dr. J. found that if a sixth of lime-water is added to milk, as advised by Dr. C., and calomel agitated with the mixture, no apparent decomposition occurs, even after several hours; nor is any such effect noticed when the proportions are as one to two. But when mixed in equal parts, the reduction to the black oxide is very evident, and quickly takes place. We need not hesitate, therefore, to give moderate quantities of lime-water in milk to patients with bowel complaints, or other affections for which we are administering calomel.

CHOLAGOGUES.—(*Lancet.*)

At a meeting of the Edinburgh Medico-Chirurgical Society, June 19th, Professor Rutherford detailed some experiments on the biliary secretion and the action of cholagogues. The subjects of experiment (dogs) were curarized, and the drugs injected into the duodenum. Physostigma is a powerful stimulant of the secretory action, its effect being neutralized by atropia; the latter by itself having no influence. Phytolacein, nitric acid, sodic benzoate, ammonic benzoate, ammonic phosphate, and sodic salicylate, act like the calabar bean, simply on the liver. Menispermin and veratrium act only on the intestinal glands. Gamboge stimulates the whole intestinal tract. Baptisin stimulates both liver and the intestinal glandular system. Tannin and potassic iodide have no effect, and plumbic acetate checks the hepatic secretion.

The true cholagogues, as mentioned above, act by stimulating the hepatic cells or their nerves. A purgative agent which only stimulates the intestinal tract, diminishes the secretion of bile by draining the portal vein.

ON SOLUTION OF IODINE IN OIL OF BITTER ALMONDS.—Blackwell. (*Phil. Med. Times August* 31, '78.) If iodine be allowed to stand in oil of bitter almonds, in proportion of one to three, for two or three months they will unite. This solution mixes freely with oils, fats, glycerine, alcohol, ethers and fluid extracts. This might be used with soap liniment for local sorbefacient use, with croton oil for counter-irritation, or with glycerine for general local use, the latter mixture leaving the skin without stain. For internal use one part of the above oil with seven parts of glycerine makes an elegant mixture, of which the dose is two drops (= $\frac{1}{20}$ gr). One part of this with 6,000 parts water has about the strength of the Saratoga Iodine spring. But the iodized oil is especially suited for admixture with cod liver oil; if to 500.0 of the latter be added 2.0 of iodized oil, 8.0 of phosphorated oil, (V. S. D.) and 0.6 of bromine a very efficient combination will be secured. The oil of bitter almonds has, in such proportions, no other than a happy effect.

ANTIDOTE FOR CARBOLIC ACID POISONING.—(*Louisville Med. News.*)

Professor Bauman has recommended, and Dr. Senftleben has used, dilute sulphuric acid as antidote to carbolic acid, and with success; the phenol and the acid combining to form phenyl-sulphuric acid, which is not poisonous. His formula was:

| | |
|---|---|
| Acid. sulph. dil. | 10 |
| Mucilag. acaciæ | 200 |
| Syrup. simp. | 20 |

Dose:—A tablespoonful every hour.

PROPYLAMINE IN CHOREA MINOR.—(*Ærtz. Intelligenzblatt.; sum. in Phil. Med. Times.*)

Pürckhauer gave propylamine in daily doses of 1.0 to 1.25. (℞ Propylamine 1.0; aq. dest. 120.0; sp. menth. pip. 25.0. Sig. tablespoonful every hour) to six persons suffering from chorea. The disease was overcome in three or four days, even where it had existed weeks. The remedy has not been used long enough to show whether such results are to be looked for in cases of rheumatic origin only.

